# Topical mitomycin C effect on swine vocal folds healing

**DOI:** 10.1016/S1808-8694(15)31015-6

**Published:** 2015-10-19

**Authors:** Paulo Antônio Monteiro Camargo, Antonio Carlos Ligocki Campos, Jorge Eduardo Fouto Matias, Daniel Zeni Rispoli, Paulo Eduardo Przysiezny, Vinícius Ribas Fonseca

**Affiliations:** a M.Sc in Surgery (Founder of the Otology and Laryngology Service at the Otorhinolaryngology Department at Angelina Caron Hospital); b Doctorate in Surgery (Professor at the Surgery Department); c Doctorate in Surgery (Assistant Professor at the Surgery Department at Ufpr); d Doctorate in Surgery (Mentor of the Pharyngostomatology and Rhinology Service at the Otorhinolaryngology Service at Angelina Caron Hospital); e M.Sc in Oro-Maxillofacial Surgery (Resident in Otorhinolaryngology at Angelina Caron Hospital); f Preceptor of Otorhinolaryngology of the Angelina Caron Hospital Otorhinolaryngology and Cervical-facial Surgery Service at Angelina Caron Hospital

**Keywords:** Mitomycin, Wound Healing, Vocal Folds, Laser Surgery, Swines

## Abstract

Several adjuvant therapies to surgery have been used to modulate the healing process of vocal folds, including topic mitomycin (MTC).

**Aim:**

to evaluate the effect of topical MTC in the healing process of vocal folds 30 days after the exeresis of a mucosal fragment with CO_2_ laser in a swine model (control group without mitomycin and study group with topical MTC), with collagen deposition measurement.

**Study type:**

prospective experimental in swine.

**Methods:**

two groups of 6 swine each were subjected to exeresis of a mucosal fragment of the vocal fold with CO_2_ laser. Immediately after the procedure MTC was applied topically for three minutes on the study group. Thirty days later the animals were slaughtered and samples of the vocal folds were collected for histological analysis, with the purpose of quantifying collagen deposition by Picrosirius Red stain.

**Results:**

the average area of total collagen in the vocal folds in the control group was 2.648,03 µm2, whereas in the study group it was 2.200,30 µm2 (p = 0.0043).

**Conclusion:**

topical application of MTC after the exeresis of a mucous fragment of vocal fold with CO_2_ laser in swine significantly decreased total collagen deposition.

## INTRODUCTION

Laryngology has made great progress with the development and the improvement of several larynx visualization techniques, especially with the surgical microscope, and more recently with video-endoscopy. Today, phonomicrosurgery has allowed surgeons to perform procedures aiming to improve vocal quality and increase recovery speed of the phonatory function^1^.

However, the unavoidable result of any surgical incision is the possibility of creating a scar, as a potential source of a problem for the speechsurgeon searching for vocal quality^2^.

Other therapies such as systemic or topical corticosteroids, cooling prior to the surgical procedure and fibrin glue, have been investigated in an attempt to help modulate the scarring process on the vocal fold, after the removal of its mucus cover^3^, ^4^.

Among the several investigated methods to modulate the scarring process, topical Mitomycin-C has proven to be an antimetabolic agent which inhibits fibroblasts proliferation when applied topically, selectively inhibiting DNA and RNA synthesis and altering protein synthesis.

The aim of this study is to evaluate scarring in pig vocal folds, after the exeresis of the mucus fragment with CO_2_ laser with and without the use of topical Mitomycin-C, and analyzing post- surgery total collagen deposition, after 30 days.

## MATERIALS AND METHODS

This prospective and experimental study was carried out at the Experimental Farm at Paraná Federal University (UFPR), located in Piraquara, state of Paraná. The study followed the Brazilian School on Animal Experimentation principles (COBEA, 1991)^5^, and the recommendations for slaughtering of experimental animals proposed by Close et al, (1997)^6^. The experiment was approved by the Institution Ethical Committee.

### Research Groups

In this experiment we used twelve Larger White pigs (6 males and 6 females) original from the Paraná Federal University farm, with an average of 10.131 grams and ages ranging from 27 to 31 days. Two randomly separated groups were formed with 6 animals each. The groups were submitted to mucus exeresis on the free rim of the left vocal fold anterior portion with CO_2_ laser. The control group did not use any topical medication on the surgical area. On the other hand, topical Mitomycin-C was applied on the experimental group for three minutes, after exeresis.

#### Surgical technique

After anesthesia, the animals were placed on their backs, on a special table for castration covered with sterile surgical dressing. The top of the table was lifted and a cushion was placed on the upper dorsal region, to increase the angle and to facilitate larynx straightening. A Storz® children laryngoscope was used, with anterior commissure angle.

The vocal folds of the pigs were visualized with a D.F. Vasconcelosâ light microscope, straight binocular with 400mm lens, and a Storz® coupled camera system. All surgical procedures were recorded in a VHS tape.

The surgical procedure started by holding the free rim of the left vocal fold anterior part, using Bouchayer Microfrance® tweezers (“heart” type). Then, using a 2 Watt CO_2_ Sharplan® laser, continuously and on the focused mode, on superpulse with 250 m microspot, the exeresis of the free rim fragment was done with 1 to 2 mm, leaving a small surgical groove on the vocal fold free rim.

After the exeresis of the fragment, cotton soaked in MMC (0.4mg/ml) was placed on the surgical wound during 3 minutes. Right after, the cotton was removed. MMC excess was removed using 0.9% saline solution.

After anesthetic recovery, the pigs were put back in their pigsties, where they received water and food, 12 hours after surgery. Food supply was given twice a day, 24 hours after surgery.

### Microscopic analysis

Thirty days after the surgical procedure, the 12 animals were submitted to the same pre-anesthetic and anesthetic procedures, followed by electrocution, according to what Close et al, (1997)^6^ proposed.

Their larynxes were removed in one piece, from the tongue to the fourth tracheal ring. An anterior longitudinal incision was made in the larynx, exposing the vocal folds, with incision of the thyroid cartilage, for the removal of all the vocal folds musculature, with the preservation of the mucus cover.

**Figure 1:**
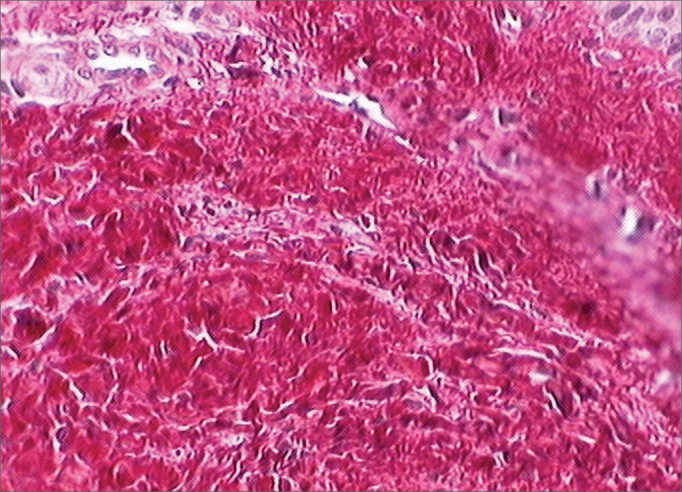
Vocal fold dyed by Sirius Red before polarization. This slide depicts the vocal fold submucosal region before polarization, and there is no distinction between cell elements and the connective tissue.

**Figure 2:**
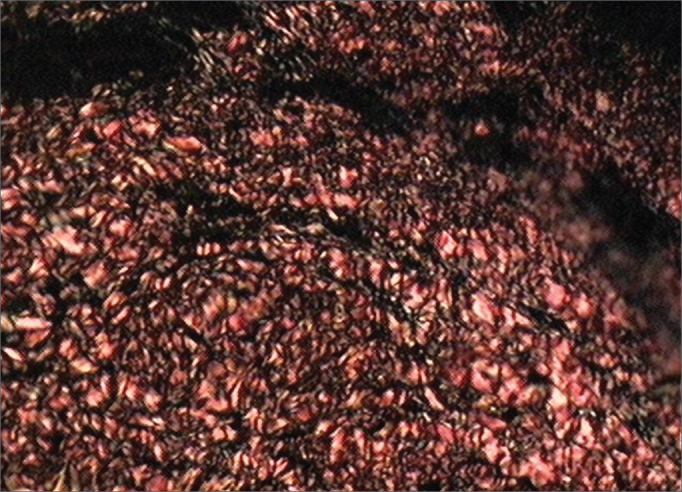
Vocal fold dyed by the Sirius Red solution after polarization. Notice the birefringent aspect: mature collagen with reddish orange dye and non-collagen substance dyed dark.

For the histological study, the Picrosirius Red solution with polarization was used. The non-collagen substance stained in black and the mature collagen in yellow, orange red and red (figures 1 and 2). For measuring the collagen deposition on the scarring process (fibroplasia) on the vocal folds mucosa surface, two histological measurements were taken in each stained sheet with Picrosirius Red after polarization.

After randomization was undone, the data was transferred to the Excel Windows® program, in order to obtain the arithmetic average. The statistical analysis was done next.

### Statistical analysis

The unpaired comparisons were done using the Wilcoxon, non-parametric test, with the value p ≤ 0.05 being the statistical significance limit.

## RESULTS

Macroscopically, all pigs had full vocal folds with physiological staining and without showing synechias after removal.

As for collagen deposition, the average collagen total area of the vocal folds in the group with exeresis of a fragment of the mucosa with laser without MMC, was of 2,648.93 μm2, whereas in the group with MMC it was of 2,200.30 μm2.

**Graph 1:**
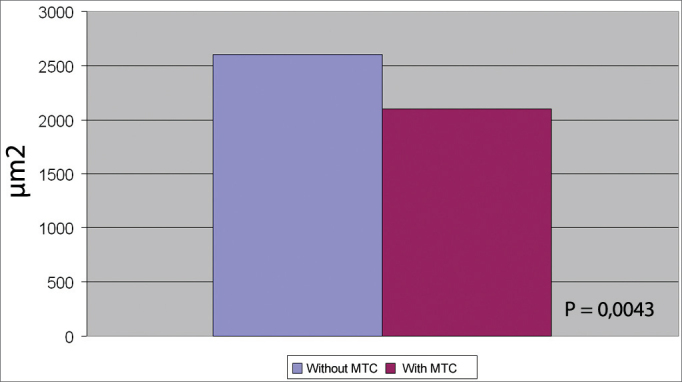
Comparison of the total collagen average on CO2 laser partial exeresis, with and without mitomycin (MMC).

In the comparison we made, the p value was of 0.0043, showing statistical difference between the collagen quantity on the group submitted to a mucosa fragment exeresis in the vocal fold with laser, both treated and not treated with MMC (graph 1).

## DISCUSSION

Even with the current techniques for mucosa and vocal ligament preservation, scars, fibrosis and consequently, stiffness are still a challenge for phonosurgeons^1^.

MMC is becoming more popular since several studies showed its efficacy in reducing post- operative fibrosis in supra glottic laryngeal stenosis^7^. Gray, Tritle (2003)^8^, noted that MMC showed a greater effect in delaying scarring, when applied right before and after damaging the tissue, suggesting that there might be an adequate time interval between the lesion formation and the application time for obtaining the proper effect. Spector et al (1999)^9^, showed significant reduction in granuloma formation and improvement in the crico-arythenoid mobility when using CO_2_ laser with topical MMC. Garrett et al (2001)^1^ showed that total collagen decrease was fixed with the use of topical MMC.

In the present study there was a statistically significant decrease of the total collagen with the use of MMC. These results suggest that the topical use of MMC when applied on the operated upon area, has an expressive effect in delaying the fibroplasia process, agreeing completely with Correa et al (1999) 10 results.

The choice of the surgical technique with CO_2_ laser, was based on Jako, Strong's studies (1972)^11^, who used it on the treatment of laryngeal papilomatosis and malignant lesions on initial stages. Currently, its indications have increased significantly with the technological evolution developed during the last years, such as the microspot decrease and the superpulse use, minimizing, as a consequence, the thermal damage on the surrounding tissues.

Santos et al (2003)^12^, compared incision on canine vocal folds made with cold instruments and with CO_2_ laser, assessing, through histological cuts, the quantity of fibroplasia. The results showed that collagen quantity on the vocal folds was greater on the group of animals submitted to lesions with cold instruments, than in those where CO_2_ laser was used. Therefore, CO_2_ laser is a safe method regarding collagen deposition, when compared to cold-bladed instruments, obviously when respecting the fundamental principles of phonomicrosurgery.

Sweat et al (1964)^13^, introduced in the histological technique the sirius red stain associated to the pricic acid (Picrosirius Red solution). Constantine, Mowry (1968)^14^, observed that the sirius red increase the birefringence of collagen fibers when studied on polarization microscope. In the current work Picrosirius Red solution was used associated with polarized microscopy, due to its great sensitivity and specificity. It is a simple and efficient method, because besides identifying the collagen fibers, it is capable of differentiating them into collagen types I, II and III through birefringence^15^, ^16^, ^17^.

Santos et al (2003)^10^, evaluated the quantity of collagen deposition on canine vocal folds, through histological cross sections stained by the Picrosirius Red solution. They concluded that this solution, together with the polarization microscopy, is better able to depict collagen fibers.

In the present study, it was showed that the use of topical MMC, when applied on the operated upon area of vocal folds, is associated to a significant reduction in collagen deposition. Future studies, with more samples and with longer follow up time, will be necessary in order to confirm these results, and to define the ideal concentration and time for applying topical MMC.

## CONCLUSION

Due to the results obtained with this experimental model in pigs, we can conclude that after 30 days of exeresis of a fragment of mucosa on the vocal folds, using CO_2_ laser, it was observed a decrease on collagen total area with the use of topical MMC applied right on top of the surgical wound, when compared to a exeresis without the use of topical MMC.
